# Diagnostic value of water-fat-separated images and CT-like susceptibility-weighted images extracted from a single ultrashort echo time sequence for the evaluation of vertebral fractures and degenerative changes of the spine

**DOI:** 10.1007/s00330-022-09061-2

**Published:** 2022-08-18

**Authors:** Georg C. Feuerriegel, Sophia Kronthaler, Christof Boehm, Martin Renz, Yannik Leonhardt, Florian Gassert, Sarah C. Foreman, Kilian Weiss, Markus Wurm, Thomas Liebig, Marcus R. Makowski, Benedikt J. Schwaiger, Dimitrios C. Karampinos, Alexandra S. Gersing

**Affiliations:** 1 Department of Radiology, Klinikum Rechts der Isar, School of Medicine, Technical University of Munich, Ismaninger Strasse 22, 81675 Munich, Germany; 2 Department of Neuroradiology, Klinikum Rechts der Isar, School of Medicine, Technical University of Munich, Munich, Germany; 3 Philips Healthcare, Hamburg, Germany; 4 Department of Trauma Surgery, Klinikum rechts der Isar, Technical University of Munich, Munich, Germany; 5 Department of Neuroradiology, University Hospital of Munich, LMU Munich, Munich, Germany

**Keywords:** Magnetic resonance imaging (MRI), Ultrashort echo time (UTE) imaging, Vertebral fractures, Bone marrow edema

## Abstract

**Objectives:**

To evaluate the performance of single-echo Dixon water-fat imaging and computed tomography (CT)–like imaging based on a single ultrashort echo time (sUTE) MR sequence for imaging of vertebral fractures as well as degenerative bone changes of the spine in comparison to conventional CT and MR sequences.

**Methods:**

Thirty patients with suspected acute vertebral fractures were examined using a 3-T MRI, including an sUTE sequence as well as short-tau inversion recovery (STIR) and T1-weighted sequences. During postprocessing, water-fat separation was performed by solving the smoothness-constrained inverse water-fat problem based on a single-complex UTE image. By removing the unwanted low-frequency phase terms, additional MR-based susceptibility-weighted-like (SW-like) images with CT-like contrast were created. Two radiologists evaluated semi-quantitative and quantitative features of fractures and degenerative changes independently and separately on CT and MR images.

**Results:**

In total, all 58 fractures were accurately detected of whom 24 were correctly classified as acute fractures with an edema detected on the water-fat-separated UTE images, using STIR and T1w sequences as standard of reference. For the morphological assessment of fractures and degenerative changes, the overall agreement between SW-like images and CT was substantial to excellent (e.g., Genant: *κ* 0.90 (95% confidence interval 0.54–1.00); AO/Magerl: *κ* 0.75 (95% confidence interval 0.43–1.00)). Overall inter-reader agreement for water-fat-separated UTE images and SW-like images was substantial to almost perfect.

**Conclusion:**

Detection and assessment of vertebral fractures and degenerative bone changes of the spine were feasible and accurate using water-fat-separated images as well as SW-like images, both derived from the same sUTE-Dixon sequence.

**Key Points:**

*• The detection of acute vertebral fractures was feasible using water-fat-separated images and CT-like images reconstructed from one sUTE sequence.*

*• Assessment of the vertebral fractures using SW-like images with CT-like contrast was found to be comparable to conventional CT.*

*• sUTE imaging of the spine can help reduce examination times and radiation exposure.*

## Introduction

Spine pathologies and degenerative changes are associated with back pain and disability and are among the greatest risk factors for morbidity and mortality worldwide [1, 2]. For adequate treatment, an accurate morphological assessment is needed to evaluate the stability of the fractured vertebral segments and the surrounding soft tissue as well as the fracture age. In clinical routine, CT provides accurate information on the osseous structures, but lacks accuracy regarding the evaluation of the bone marrow and soft tissue components. Therefore, additional MR imaging is often performed [3–5], yet, combined imaging with CT and MRI is associated with overall increased costs and duration of the diagnostic evaluation [6]. Furthermore, CT causes additional radiation exposure to the patient. Acquisition of all diagnostic information on osseous pathologies as well as soft tissue with one examination would therefore be highly beneficial for the patient. Several approaches using high-resolution MR sequences with bone-specific sequences have been previously suggested for imaging of osseous structures: CT-like images, derived from a 3D T1 spoiled gradient-echo sequence (GRE) and derived from UTE images, previously showed high agreement and comparable results to CT when assessing vertebral fractures and degenerative changes of the spine [7]. Furthermore, 3D UTE and zero echo time (ZTE) imaging were used to assess cortical and trabecular bone [8] and previously susceptibility-weighted MRI also showed to be reliable and accurate regarding the evaluation of vertebral fractures [9]. Most recently, different promising deep learning–based approaches were proposed, including the creation of “synthetic” or “pseudo” CTs from MRI using convolutional neuronal networks (CNN) or generative adversarial networks (GAN) [10, 11].

For the assessment of the fracture age, STIR- and T1-weighted sequences are usually acquired to identify edema-equivalent bone marrow changes, which are considered to be highly specific for bone marrow edema after an acute trauma [12, 13]. Recent studies also suggested the use of a T2-weighted Dixon sequence for the detection of occult fractures and the differentiation of malignant versus benign vertebral fractures in order to shorten the examination duration [14, 15]. Nevertheless, accurate examination of the spine with regular morphological sequences or with a combination of the sequences mentioned previously would take at least 10 to 15 min and require several MR sequences. More recent approaches described methods of fat and water separation or fat suppression for UTE images, which are able to assess the signal of tissues with short T_2_^*^ relaxation times [16–18]. However, at least two echoes need to be acquired to suppress fat in the UTE image, which can prolong the scan time. Similarly, to separate water and fat, conventional Dixon imaging was combined with UTE imaging [19]. However, several echoes need to be acquired and UTE-Dixon has its limitations since it does not take the short T_2_^*^ decay of water signal into account [16]. Single-echo Dixon (sTE-Dixon) methods have been used to reduce scan time, yet an additional reference scan is needed to remove the unwanted phase terms [16, 20, 21]. Recently a new method was proposed by using ultrashort echo time cones double echo steady state (UTE-Cones-DESS) imaging which showed high morphological contrast for tissue with short T_2_, again requiring the acquisition of two complex signals to solve the background phase terms in postprocessing which prolonged the scan time [17].

The purpose of this study was to evaluate the diagnostic value of a newly developed method [22] that simultaneously performs single-TE Dixon and SW-like imaging with CT-like contrast based on a single-echo UTE spoiled gradient echo acquisition for the evaluation of acute vertebral fractures and degenerative changes of the spine.

## Materials and methods

### Patient selection

The study was approved by our institutional review board (Ethics Commission of the Medical Faculty, Technical University of Munich, Germany; Ethics proposal number 537/20 S-KH). Informed consent was obtained from all study participants prior to inclusion. Participants which were admitted to the emergency unit were recruited from November 2018 until September 2019. In total, 30 patients (65.3 ± 17.5 years, 19 women) with suspected acute thoracolumbar vertebral fracture were included into the study. All patients received a CT scan of the spine as part of the routine clinical diagnostic work flow and received a MRI within 3 days.

### CT scan

Each patient received a CT scan of the thoracolumbar spine using either a Philips IQon Spectral CT scanner or a Siemens Somatom Definition AS+ scanner. Clinical scan parameters were set according to the clinical routine: collimation, 0.6 mm; pixel spacing, 0.4/0.3 mm; pitch factor, 0.8/0.9; tube voltage (peak), 120 kV; and modulated tube current, 102–132 mA. Images were acquired in axial orientation and reformatted in sagittal and coronal orientation using a bone-specific convolution kernel (170H/YB, 3-mm slices).

### MR imaging

For MR imaging, a standard spine protocol was used including a sagittal T1-weighted sequence with turbo spin echo (T1w TSE) *and* a sagittal STIR sequence. To measure the signal of tissues with short T_2_* values, a 3D UTE stack-of-stars sequence was employed [23, 24] with a non-selective rectangular RF pulse. The RF excitation was followed by a variable-duration slice encoding gradient. The following scan parameters were used: echo time 0.14 ms, repetition time 6.3 ms, flip angle 5°, field of view (FOV) 250 × 250 × 279 mm^3^, voxel size (acquired) 0.45 × 0.45 × 3 mm^3^, voxel size (reconstructed) 0.28 × 0.28 × 0.75 mm^3^ and acquisition time 6.3 ± 0.23 min, ramp length 0.08 ms, max. gradient strength 15.04 mT/m, sampling dwell time 3.12 μs with 568 samples, acquisition window 1.77 ms, 945 number of spokes, with radial percentage of 85%, partial Fourier with a factor of 0.6 in slice direction. Image reconstruction was performed offline and gradient imperfections were corrected by means of a gradient impulse response function [23]. During the reconstruction, the images were Fourier interpolated from the acquired resolution to the reconstruction resolution. All participants were examined using a 3-Tesla MR scanner (Ingenia; Philips Healthcare) with dedicated 16-channel anterior and posterior body coils (dStream Torso coil, Philips Healthcare).

### Postprocessing sUTE-Dixon

For postprocessing of the single-echo UTE images, sUTE-Dixon water-fat separation was performed. The algorithm solves the water and fat inverse problem while simultaneously removing unwanted phase terms in the UTE phase. The unwanted phase terms were mainly composed of phase due to *B*_0_ inhomogeneities, eddy currents, signal delays in the receiver chains, and the *B*_1_ transmit/receive phase. Due to the smoothly and slowly varying nature of these unwanted phase terms, an iterative and smoothness constrained approach was used as done previously [22]. The newly developed algorithm solves the smoothness-constrained non-linear inverse water-fat problem while at the same time removing the unwanted low-frequency phase terms. All sUTE-Dixon processing computations were performed offline in Python on the graphics card of a workstation with GPU 24GiB RAM, 24-core CPU (Intel Xeon Gold), and 768 GB memory. In average, the water-fat separation of a full UTE spine data set took 160 s [22].

### SWI-like processing

SW-like images were created by using the corrected UTE phase from the sUTE-Dixon processing. Based on the corrected UTE phase, phase masks were generated and scaled between 0 and 1 to increase bone contrast. The phase masks were then *n* = 1–5 times multiplied with the magnitude of the original UTE image to achieve an improved contrast *S*′_n_. In order to achieve a CT-like contrast with high “bone-like” signal, the SW-like images were inverted.

### Image analysis

CT and MR images were read by two radiologists (M.R., board-certified radiologist with over 7 years of experience, and Y.L. with 4 years of experience in musculoskeletal radiology). The images were read individually and independently in a random order and blinded to clinical information and any other imaging data. The different MR sequences and CT images were read with at least 8 weeks in between readings, respectively. For intra-reader reproducibility, 10 patients were assessed once again after 8 weeks by both radiologists. Image analyses were performed on a PACS work station certified for clinical use (IDS7 21.2, Sectra).

### Quantitative measurements

The images were assessed for the presence and exact location of vertebral fractures. For the evaluation of the fracture age, STIR and water-separated sUTE images were analyzed for the presence of edema-like bone marrow signal. T1-weighted images and fat-separated sUTE images were evaluated for a corresponding signal drop. Additionally, UTE-based SW-like images and CT were assessed for the visibility of fracture lines and contrast of the vertebrae using a 5-point Likert scale (1 = poor, 2 = below average, 3 = fair, 4 = good, 5 = excellent). Morphological features of the spine were analyzed and graded according to previously described clinical scores [7]. Fractures were classified according to AO/Magerl [25, 26], and the differentiation of acute and chronic vertebral fractures was performed as previously defined [27]. The height of the anterior and posterior vertebral body in the mid-sagittal plane was measured and the height loss was classified according to Genant et al [28]. Degenerative changes were evaluated in two adjacent non-fractured vertebrae, including the intervertebral disc height [29], presences of Schmorl’s nodes, anteroposterior (AP) diameter of intervertebral foramina [30], and spondylolisthesis. Furthermore, the sclerosis-like bone signal adjacent to one or both vertebral endplates, osteophyte formation [31], and facet joint degeneration [32] were assessed. Overall diagnostic image quality was graded using a 5-point Likert scale.

### Statistics

The data were analyzed using IBM SPSS Statistics for Windows, version 27.0 (IBM Corp.). All statistical tests were performed two-sided and a level of significance (*α*) of 0.05 was used. Agreement of ordinal scaled parameters was assessed using weighted Cohen’s *κ* [33]. The agreement of numerical data was evaluated with intra-class correlation coefficients (ICC). The inter- and intra-observer reliabilities were also calculated using Cohen’s kappa und ICC, respectively [34]. Descriptive statistics were performed using paired *t*-tests (for numeric variables) and McNemar’s tests (for binary categorical variables).

## Results

### Fracture characteristics and image quality

The overall mean image quality for both readers measured with a 5-point Likert scale was 4.1 ± 0.81 for the water-separated sUTE images and 4.3 ± 0.63 for the fat-separated sUTE images. Overall image quality of the SW-like images was rated good (mean 4.38 ± 0.70), with a slight difference depending on the phase masks applied (range 4.10–4.58, *n* = 1–5; Table 3).

Overall, 58 fractures were detected using CT as standard of reference. The most affected vertebra was L5 with 22 % of the fractures. Twenty-four out of 58 fractures were classified as acute fractures due to an edema-like signal identified in the morphological MR images. The majority (83%) of the fractures were classified as AO3.

### Diagnostic agreement of STIR and T1-weighted images and water-/fat-separated sUTE images

**Fig. 1:**
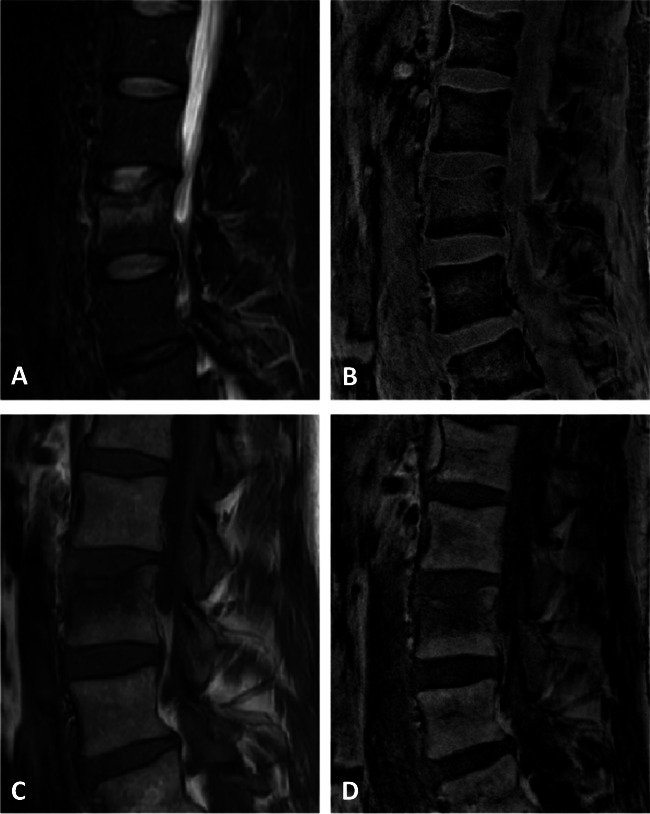
Standard short-tau inversion recovery sequence image (STIR) showing a bone marrow edema of an acute fracture of the second lumbar vertebra (**A**). UTE-derived water-separated image showing an edema equivalent to the STIR sequence (**B**). Standard T1-weighted sequence image (**C**) and correlating to UTE-derived fat-separated image (**D**) both showing a signal reduction within the fractured vertebra

**Fig. 2:**
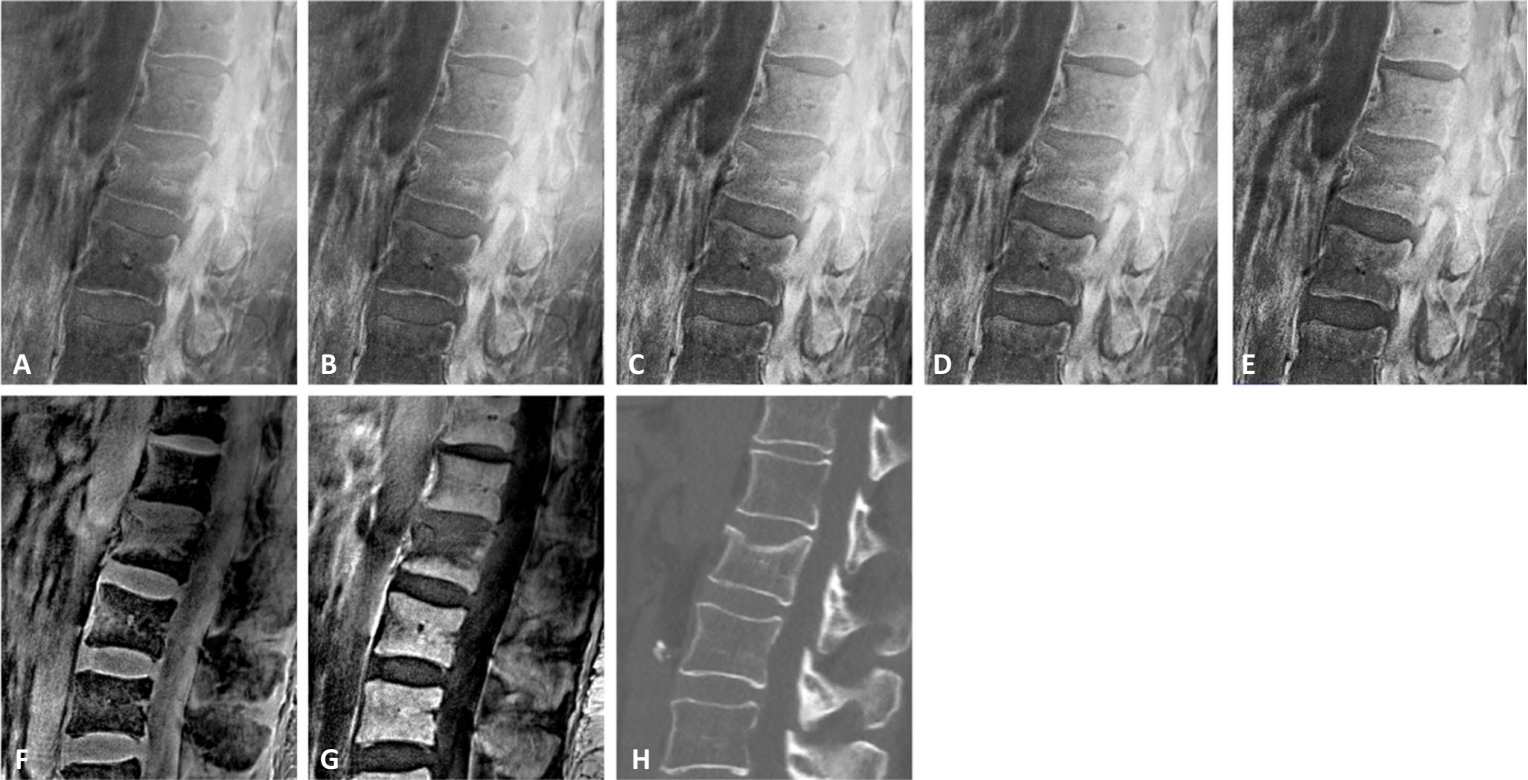
UTE-derived SW-like images with CT-like contrast and corresponding water- and fat-separated images (**A**–**G**) showing an acute compression fracture in L2. For postprocessing of SW-like images, the input UTE phase was corrected and unwanted phase terms were removed. Afterwards phase masks were applied to the UTE magnitude image to increase the contrast of areas with negative phase. Note the increasing contrast of the vertebrae with the increasing number of phase masks applied (**A** *S*′_1_ with phase mask applied once and **E** *S*′_5_ with phase mask applied 5 times), in comparison to the conventional CT scan (**H**). **F** and **G** show the sUTE-Dixon water- and fat-separated images, respectively

**Fig. 3:**
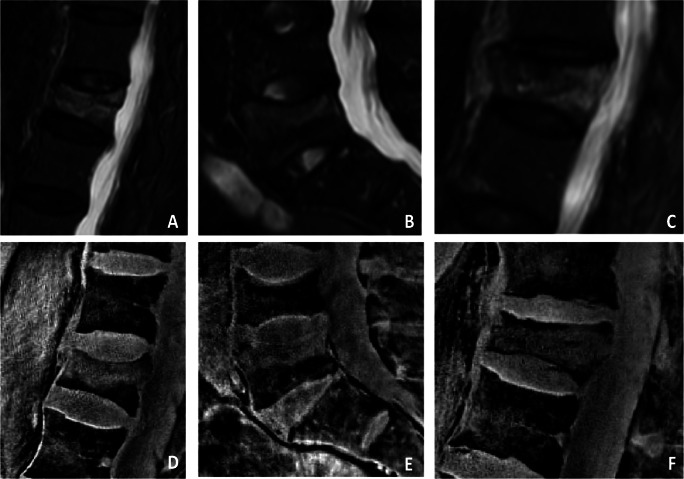
STIR images of acute vertebral fractures showing hyperintense edema-like signal in L1 (**A**), L5 (**B**), and L3 (**C**). Corresponding water-separated sUTE images showing an equivalent bone marrow edema within the fractured vertebrae (**D**–**F**)

Edema-like signal was detected in all 24 acute fractures by both readers using the water-separated sUTE images (readers 1 and 2: *κ* 1.00 (95% confidence interval 1.00–1.00)) (Figs. 1 and 3), using the morphological sequences as standard of reference. A corresponding signal drop was identified on the fat-separated images. Of the 34 non-acute vertebral fractures, two fractures were falsely classified as acute fractures due to hyperintense signal adjacent to the fracture in the water-separated sUTE images. The overall agreement for fracture classification was substantial to almost perfect (AO Magerl, *κ* 0.87 (95% confidence interval 0.36–1.00); Genant classification, *κ* 0.89 (95% confidence interval 0.48–1.00)). The overall agreement regarding the evaluation of the fracture age was excellent, *κ* 0.77 (95% confidence interval 0.33–1.00), and for degenerative changes substantial to almost perfect, *κ* 0.90 (95% confidence interval 0.15–1.00, Table 1) (Figs. 2 and 3).

**Table 1:** Agreement between the standard MR sequences STIR and T1w and the UTE-based water- and fat-separated images.

Parameters	STIR and water-separated UTE		T1w and fat-separated UTE	
	Reader 1	Reader 2	Reader 1	Reader 2
Fractures				
BME^1^	1.00 [1.00–1.00]	1.00 [1.00–1.00]	1.00 [1.00–1.00]	1.00 [1.00–1.00]
Genant classification^1^	0.93 [0.80–1.00]	0.74 [0.48–0.94]	1.00 [1.00–1.00]	0.88 [0.70–1.00]
AO Magerl^1^	0.92 [0.72–1.00]	0.91 [0.68–1.00]	0.92 [0.72–1.00]	0.74 [0.36–1.00]
Visibility of fracture morphology^1^	0.90 [0.73–1.00]	0.49 [0.21–0.72]	0.83 [0.64–1.00]	0.83 [0.64–1.00]
Vertebral contrast^1^	0.85 [0.63–1.00]	0.84 [0.59–1.00]	0.73 [0.49–0.94]	0.94 [0.81–1.00]
Image quality^1^	0.88 [0.71–1.00]	0.81 [0.52–1.00]	0.84 [0.58–1.00]	0.65 [0.00–1.00]
Fracture age^1^	0.86 [0.66–1.00]	0.78 [0.46–1.00]	0.71 [0.33–1.00]	0.73 [0.41–0.94]
Degenerative changes				
Anterior vertebral body height^2^	0.87 [0.42–0.96]	0.85 [0.15–0.96]	0.99 [0.98–0.99]	0.97 [0.94–0.98]
Posterior vertebral body height^2^	0.89 [0.44–0.96]	0.91 [0.25–0.98]	0.97 [0.94–0.99]	0.93 [0.84–0.97]
Anterior disc height^2^	0.99 [0.93–0.99]	0.79 [0.37–0.92]	0.98 [0.97–0.99]	0.98 [0.98–0.99]
Posterior disc height^2^	0.99 [0.99–0.99]	0.71 [0.37–0.87]	0.87 [0.72–0.94]	0.99 [0.99–0.99]
Neuroforamina diameter^2^ (AP)^2^	0.96 [0.91–0.98]	0.90 [0.77–0.96]	0.95 [0.89–0.99]	0.96 [0.91–0.98]
Facet joint degeneration^1^	0.73 [0.49–0.94]	0.73 [0.48–0.94]	0.84 [0.62–1.00]	0.84 [0.64–1.00]

^1^Weighted Cohen’s kappa (*κ*), ^2^interclass correlation coefficient; data are given with 95% confidence interval

### Diagnostic agreement of CT and SW-like images

**Fig. 4:**
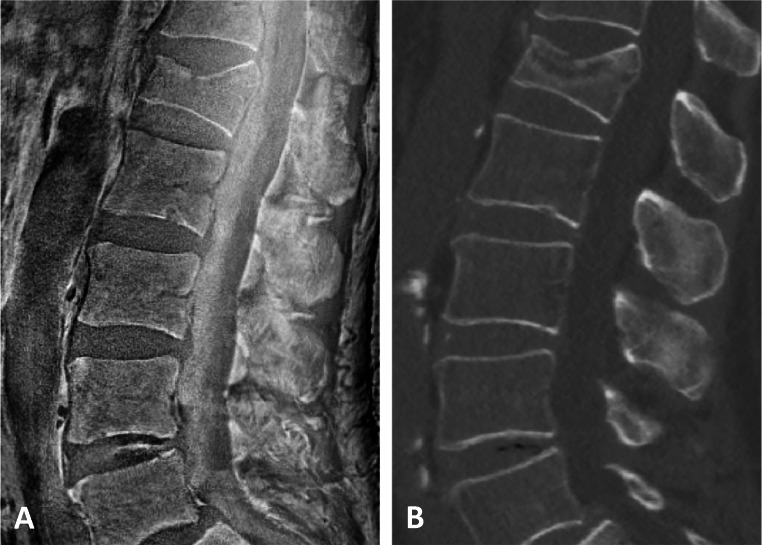
UTE-derived SW-like images with CT-like contrast of an acute compression fracture of the 1st lumbar vertebra (**A**). The fracture can be evaluated equivalent compared to the conventional CT scan (**B**)

All vertebral fractures were accurately detected using SW-like images with CT-like contrast by both readers (*N* = 58, *κ* 1.00 (95% CI 1.00–1.00) for both readers, Figs. 2 and 4) (Table 2). The mean image quality of the CT-like images increased with the number of phase masks that were applied, with the mean image quality ranging from 4.11 to 4.41 for reader 1 and from 4.46 to 4.58 for reader 2 (Table 3). Similarly, the contrast of the vertebrae to the surrounding soft tissue which increased with the number of phase masks applied ranging from 3.76 to 4.42 for reader 1 and from 3.77 to 4.77 for reader 2. A slight decrease was seen in the visibility of the fracture morphology in reader 1 (range from 2.53 to 2.20 for reader 1 and from 2.54 to 3.54 for reader 2).

**Table 2:** Agreement between the UTE-based SW-like images with five phase masks applied and conventional multidetector CT

Parameters	UTE-based SW-like images and CT	
	Reader 1	Reader 2
Fractures		
Genant classification^1^	1.00 [1.00–1.00]	0.80 [0.54–1.00]
AO Magerl^1^	0.62 [0.27–0.92]	0.79 [0.43–1.00]
Visibility of fracture morphology^1^	0.77 [0.54–0.94]	0.88 [0.70–1.00]
Vertebral contrast^1^	0.62 [0.20–1.00]	0.83 [0.00–1.00]
Image quality^1^	0.85 [0.61–1.00]	0.92 [0.67–1.00]
Fracture age^1^	0.64 [0.34–0.87]	0.77 [0.47–1.00]
Degenerative changes		
Anterior vertebral body height^2^	0.83 [0.60–0.93]	0.82 [0.60–0.93]
Posterior vertebral body height^2^	0.95 [0.89–0.98]	0.93 [0.83–0.97]
Anterior disc height^2^	0.69 [0.31–0.84]	0.91 [0.77–0.96]
Posterior disc height^2^	0.81 [0.56–0.92]	0.90 [0.77–0.95]
Neuroforamina diameter^2^ (AP)^2^	0.71 [0.31–0.88]	0.95 [0.88–0.98]
Facet joint degeneration^1^	0.94 [0.80–1.00]	0.82 [0.61–1.00]
Sclerosis^1^	0.89 [0.51–1.00]	0.75 [0.27–1.00]
Osteophytes^1^	0.77 [0.45–1.00]	0.85 [0.60–1.00]

^1^Weighted Cohen’s kappa (*κ*), ^2^interclass correlation coefficient; data are given with 95% confidence interval

**Table 3:** Evaluation of the UTE-based SW-like images depending on the number of phase masks applied (*n* = 1 to *n* = 5)*

	Reader 1			Reader 2		
	Image quality	Visibility of the fracture morphology	Vertebral contrast	Image quality	Visibility of the fracture morphology	Vertebral contrast
SWI *n* = 1	4.11 ± 0.90	2.53 ± 0.70	3.76 ± 0.90	4.46 ± 0.70	2.54 ± 0.80	3.77 ± 0.77
SWI *n* = 2	4.19 ± 0.80	2.73 ± 0.70	3.96 ± 0.70	4.42 ± 0.80	2.54 ± 0.80	3.92 ± 0.70
SWI *n* = 3	4.23 ± 0.70	2.30 ± 0.80	3.90 ± 0.90	4.46 ± 0.70	2.39 ± 0.90	4.04 ± 0.80
SWI *n* = 4	4.38 ± 0.70	2.20 ± 0.70	4.35 ± 0.70	4.54 ± 0.60	2.39 ± 0.90	4.39 ± 0.90
SWI *n* = 5	4.41 ± 0.70	2.20 ± 0.70	4.42 ± 0.80	4.58 ± 0.70	3.54 ± 1.12	4.77 ± 0.50
CT	4.92 ± 0.40	4.00 ± 1.10	4.92 ± 3.20	4.60 ± 0.70	4.90 ± 0.40	3.90 ± 1.17

*Mean ± standard deviation

AO classification of the fractures was graded identical for SW-like images with 5 phase masks applied by both readers compared to CT (readers 1 and 2: *κ* 1.00 (95% confidence interval 1.00–1.00, Table 2)). For quantitative parameters describing degenerative changes, the agreement was excellent, ranging between ICC 0.69 (95% CI 0.31–0.89, reader 1) for anterior disc height and 0.95 (95% CI 0.88–0.98, reader 2) for neuroforaminal diameter. The agreement for ordinal-scale parameters ranged from substantial (sclerosis; *κ* 0.75 (95% CI 0.27–1.00, reader 2)) to almost perfect (facet joint degeneration; *κ* 0.94 (95% CI 0.80–1.00, reader 1)).

### Inter-reader agreement for water- and fat-separated sUTE images and SW-like images

Inter-reader agreement for water- and fat-separated sUTE images was excellent ranging from *κ* 0.62 (95% CI 0.35–0.84) for the contrast of the vertebrae to perfect (*κ* 1.00 (95% confidence interval 1.00–1.00)) for classification of the fracture age and edema-like signal detection (Table 4). The inter-reader agreement for the SW-like images ranged from *κ* 0.82 (95% CI 0.59–1.00) for the fracture age to *κ* 1.00 (95% CI 1.00–1.00) for the fracture classification (AO/Genant) and detection of osteophytes (Table 4).

**Table 4:** Inter-reader reliability between the findings of reader 1 and reader 2

Parameter	STIR	T1w	Water	Fat	SWI *n* = 5	CT
Fractures						
BME^1^	1.00 [1.00–1.00]	0.87 [0.47–1.00]	1.00 [1.00–1.00]	0.87 [0.52–1.00]		
Genant classification^1^	0.88 [0.69–1.00]	0.77 [0.55–0.94]	0.94 [0.81–1.00]	0.82 [0.62–1.00]	1.00 [1.00–1.00]	0.81 [0.58–1.00]
AO Magerl^1^	0.85 [0.61–1.00]	1.00 [1.00–1.00]	0.70 [0.41–0.93]	0.70 [0.39–0.93]	1.00 [1.00–1.00]	1.00 [1.00–1.00]
Visibility of fracture morphology^1^	0.69 [0.46–0.89]	0.72 [0.42–0.94]	0.66 [0.43–0.85]	0.85 [0.66–1.00]	0.80 [0.55–1.00]	0.83 [0.65–1.00]
Vertebral contrast^1^	0.70 [0.42–0.93]	0.81 [0.58–1.00]	0.62 [0.35–0.84]	0.83 [0.61–1.00]	0.87 [0.68–1.00]	0.78 [0.31–1.00]
Image quality^1^	0.88 [0.68–1.00]	1.00 [1.00–1.00]	0.83 [0.62–1.00]	0.93 [0.77–1.00]	0.92 [0.73–1.00]	0.84 [0.15–1.00]
Fracture age^1^	0.84 [0.60–1.00]	1.00 [1.00–1.00]	1.00 [1.00–1.00]	0.89 [0.71–1.00]	0.82 [0.59–1.00]	0.87 [0.66–1.00]
Degenerative changes						
Anterior vertebral body height^2^	0.99 [0.96–0.99]	0.97 [0.95–0.98]	0.99 [0.98–1.00]	0.99 [0.99–0.99]	0.96 [0.92–0.98]	0.99 [0.98–0.99]
Posterior vertebral body height^2^	0.98 [0.98–0.99]	0.98 [0.97–0.99]	0.98 [0.96–0.99]	0.98 [0.97–0.99]	0.98 [0.96–0.99]	0.99 [0.98–0.99]
Anterior disc height^2^	0.97 [0.95–0.99]	0.98 [0.95–0.99]	0.89 [0.74–0.95]	0.98 [0.97–0.99]	0.97 [0.94–0.99]	0.98 [0.96–0.99]
Posterior disc height^2^	0.95 [0.90–0.98]	0.95 [0.91–0.98]	0.86 [0.65–0.94]	0.95 [0.89–0.98]	0.94 [0.86–0.97]	0.96 [0.91–0.98]
Neuroforamina diameter^2^ (AP)	0.97 [0.94–0.99]	0.95 [0.88–0.98]	0.83 [0.63–0.92]	0.99 [0.97–0.99]	0.94 [0.86–0.97]	0.96 [0.92–0.99]
Facet joint degeneration^1^	1.00 [1.00–1.00]	0.75 [0.55–0.95]	1.00 [1.00–1.00]	0.89 [0.71–1.00]	0.90 [0.74–1.00]	1.00 [1.00–1.00]
Osteophytes^1^	0.74 [0.49–0.94]	0.95 [0.83–1.00]	0.65 [0.41–0.87]	1.00 [1.00–1.00]	1.00 [1.00–1.00]	1.00 [1.00–1.00]
Schmorl nodes^1^	1.00 [1.00–1.00]	0.89 [0.59–1.00]	0.87 [0.57–1.00]	0.83 [0.43–1.00]	0.87 [0.47–1.00]	0.83 [0.54–1.00]

^1^Weighted Cohen’s kappa (*κ*)

^2^Interclass correlation coefficient

*Data are given with 95% confidence interval

### Intra-reader agreement for water- and fat-separated sUTE images and SW-like images

For intra-reader reliability, both readers assessed the images of 10 patients after at least 8 weeks separately, independently and blinded to all clinical information. The intra-reader agreement was overall substantial to almost perfect (range *κ* 0.71 to 1.00) for both readers (Table 5). All acute fractures were once more accurately identified on the water-separated UTE images (*N* = 24, *κ* 1.00 (95% CI 1.00–1.00) for both readers).

**Table 5:** Intra-reader reliability of reader 1 and reader 2 for water- and fat-separated UTE images and SW-like images with CT-like contrast

Parameter	Reader 1		Reader 2		SW-like images	
	Water	Fat	Water	Fat	Reader 1	Reader 2
Fractures						
BME^1^	1.00 [1.00–1.00]	1.00 [1.00–1.00]	1.00 [1.00–1.00]	1.00 [1.00–1.00]		
Genant classification^1^	1.00 [1.00–1.00]	1.00 [1.00–1.00]	1.00 [1.00–1.00]	1.00 [1.00–1.00]	1.00 [1.00–1.00]	1.00 [1.00–1.00]
AO Magerl^1^	0.85 [0.47–1.00]	0.85 [0.50–1.00]	0.79 [0.30–1.00]	0.80 [0.30.–0.99]	1.00 [1.00–1.00]	1.00 [1.00–1.00]
Visibility of fracture morphology^1^	0.84 [0.42–1.00]	0.71 [0.23–1.00]	0.71 [0.33–1.00]	0.71 [0.20–1.00]	0.83 [0.46–1.00]	0.89 [0.56–1.00]
Vertebral contrast^1^	0.84 [0.41–1.00]	0.72 [0.30–1.00]	0.83 [0.41–1.00]	0.86 [0.49–1.00]	0.85 [0.51–1.00]	0.84 [0.54–1.00]
Image quality^1^	0.82 [0.46–1.00]	0.83 [0.46–1.00]	0.85 [0.50–1.00]	0.84 [0.44–1.00]	0.84 [0.50–1.00]	0.83 [0.33–1.00]
Fracture age^1^	1.00 [1.00–1.00]	1.00 [1.00–1.00]	1.00 [1.00–1.00]	1.00 [1.00–1.00]	1.00 [1.00–1.00]	1.00 [1.00–1.00]
Degenerative changes						
Anterior vertebral body height^2^	0.98 [0.95–0.99]	0.99 [0.97–0.99]	0.97 [0.90–0.99]	0.99 [0.96–0.99]	0.98 [0.94–0.99]	0.99 [0.97–0.99]
Posterior vertebral body height^2^	0.98 [0.94–0.99]	0.99 [0.94–0.99]	0.98 [0.91–0.99]	0.96 [0.85–0.99]	0.98 [0.90–0.99]	0.99 [0.94–0.99]
Anterior disc height^2^	0.97 [0.86–0.99]	0.97 [0.88–0.99]	0.99 [0.95–0.99]	0.98 [0.94–0.99]	0.97 [0.88–0.99]	0.98 [0.93–0.99]
Posterior disc height^2^	0.94 [0.76–0.98]	0.98 [0.90–0.99]	0.96 [0.83–0.99]	0.94 [0.79–0.98]	0.97 [0.90–0.99]	0.95 [0.80–0.98]
Neuroforamina diameter^2^ (AP)	0.96 [0.81–0.99]	0.98 [0.92–0.99]	0.90 [0.62–0.98]	0.96 [0.82–0.99]	0.98 [0.93–0.99]	0.99 [0.96–0.99]
Facet joint degeneration^1^	1.00 [1.00–1.00]	1.00 [1.00–1.00]	1.00 [1.00–1.00]	1.00 [1.00–1.00]	1.00 [1.00–1.00]	1.00 [1.00–1.00]
Osteophytes^1^	1.00 [1.00–1.00]	1.00 [1.00–1.00]	1.00 [1.00–1.00]	1.00 [1.00–1.00]	1.00 [1.00–1.00]	1.00 [1.00–1.00]
Schmorl nodes^1^	1.00 [1.00–1.00]	1.00 [1.00–1.00]	1.00 [1.00–1.00]	1.00 [1.00–1.00]	1.00 [1.00–1.00]	1.00 [1.00–1.00]

^1^Weighted Cohen’s kappa (*κ*)

^2^Interclass correlation coefficient

*Data are given with 95% confidence interval

## Discussion

In this study, we showed that water- and fat-separated Dixon imaging as well as SW-like imaging with CT-like contrast is feasible using only one single UTE scan of the spine. Detection and assessment of an edema-like signal in acute vertebral fractures was feasible and accurate using water-separated sUTE images compared to standard morphological MR sequences. A substantial to almost perfect agreement was found for the assessment of degenerative bone changes using fat- and water-separated images compared to standard MR sequences. The SW-like images with CT-like contrast, which were reconstructed from the same sUTE, showed an excellent agreement for the morphological assessment and accuracy in the detection of vertebral fractures and degenerative changes compared to conventional CT.

In clinical routine, patients usually receive a conventional CT and an additional MRI with multiple sequences to assess the fracture age and degenerative changes of the spine. Acquiring all diagnostic information for the assessment of vertebral fractures in one examination/sequence may therefore reduce the overall examination duration and costs as well as reduce the radiation exposure of the patient caused by the CT scan. Additionally, acquisition time was about 6.3 ± 0.23 min for the presented sequence, which is overall significantly shorter compared to the combined examination duration of CT and MRI with multiple sequences. An overall shorter examination duration may be beneficial for patients that experience severe pain, which may be the case in patients with vertebral fractures, or suffer from claustrophobia. Therefore, single UTE reconstructed water- and fat-separated images and SW-like images may be a useful alternative to the standard MRI and CT when assessing vertebral fractures or degenerative changes of the spine.

To our knowledge, this is the first study using a single TE UTE scan of the spine for the reconstruction of water- and fat-separated images and simultaneously acquired SW-like images with CT-like contrast in a clinical study cohort, by solving the smoothness-constrained non-linear inverse water-fat problem while at the same time removing the unwanted low-frequency phase terms. In a recent study, single-echo Dixon processing in UTE DESS imaging was used for patients with osteoarthritis of the knee and was able to show a high contrast for tissue with short T_2_ and sufficient fat saturation [17]. In contrast to our study, two complex signals were used to directly solve for background contributions. The approach presented in this study used an iterative non-linear optimization approach to remove the unwanted low-frequency phase terms based on a single complex signal.

Furthermore, in our study, data postprocessing of the sUTE images was fully automatic by removing the unwanted low-frequency phase terms, using an iterative non-linear optimization approach which is an advantage compared to filtering methods. The processing was performed offline, however could potentially be performed on the scanner, since no manual fine-tuning is needed.

Dixon imaging has also been used as an alternative to standard MR for the differentiation of malignant and benign vertebral fractures [15], but the proposed six-echo Dixon method mainly assesses the fat fraction and has its limitation with regard to the differentiation of acute and old vertebral fractures. Another advantage of the methodology proposed in this study is the simultaneous acquisition of SW-like images with CT-like contrast. SW-like images showed a substantial to almost perfect agreement for the classification and detection of vertebral fractures compared to conventional CT. Distance measurements as well as the evaluation of sclerosis were excellent and comparable to CT. A previous study demonstrated the possibility to detect vertebral fractures on susceptibility-weighted MRI [9]. Although the acquired slice thickness was comparable to our study, the previous study was performed using a 1.5-T MR scanner, which consequently reduces the image quality and might reduce the diagnostic information.

For MR imaging of osseous parts of the spine, the use of a T_1_ GRE sequence was recently suggested, which was able to show a performance similar to the performance of a CT [7] and therefore showed accurate results for the detection of vertebral fractures.

Yet, compared to UTE imaging, ligaments, calcifications, and other tissues with short T_2_ appear brighter on the intensity inverted T_1_ GRE images which could make the identification and differentiation of certain pathologies more challenging, e.g., in cases presenting with ankylosing spondylitis. The current gold standard for the diagnosis of spine fractures and degenerative changes usually includes several morphologic MR sequences (e.g., STIR and T1-weighted sequences) as well as in most cases a CT for the evaluation of the fracture itself. Using the proposed image reconstructions of the UTE sequence, both SW-like images with CT-like contrast and water-fat-separated images can be reconstructed from one single UTE sequence, which consequently may save scan time, since in theory no additional sequences or examinations would be needed. The reconstructions are performed on a separate server.

There are some limitations to this study. Although the radial acquisition of the images made the scan less motion sensitive, the acquisition time of approximately 6.3 min itself creates a certain susceptibility to motion artifacts. Furthermore, the UTE was susceptible to slight fat blurring due to the radial k-space trajectory which depends on the readout bandwidth and the in-plane resolution of the scan protocol. In some cases, a slight hyperintense signal was seen in the water-separated sUTE images adjacent to the fractures which was presumably caused by the vertebral venous plexus and was misinterpreted in two cases as an acute fracture. The signal of the presumed vertebral venous plexus usually appeared to be weak hyperintense with a lower signal than the signal of an acute bone marrow edema caused by a fracture. Yet, when assessed simultaneously with the SW-like images, the vertebral venous plexus can be differentiated in the vast majority of the cases from an acute fracture due to the weaker signal hyperintensity compared to edema and the missing fracture morphology. This needs to be taken into consideration when interpreting the images and for fracture assessment the fat- and water-separated images as well as SW-like images should be evaluated simultaneously to avoid false-positive results.

Another limitation was the use of phase information in the weighting of the SWI magnitude, which causes not only the osseous structures to be weighted but also parts with high fat content. Therefore, SW-like images comprised both susceptibility and chemical shift effects. In this study, an increased contrast of the vertebrae to the surrounding soft tissue was seen with increasing phase masks applied. Inversely a slight but non-significant decrease in the visibility of the thin cortical layer and fracture lines was detected most likely due to the increasing chemical shift effects. This needs to be considered when evaluating the SW-like images. Effects on the contrast of trabecular bone were not assessed as the primary focus was on the fracture detection and differentiation. Patients with metallic implants were not included in the study due to artifacts in 3-T scanners. Nevertheless, due to the half-radial readout of UTE sequences, UTE imaging is known to be less affected by artifacts due to implants and strong *B*_0_ inhomogeneities when compared to Cartesian or radial imaging. The use of artifact reduced UTE sequences on 3-T and 1.5-T MR systems needs to be assessed in the future.

## Conclusion

In summary, detection and age assessment of vertebral fractures as well as the morphological assessment of fractures and degenerative bone changes of the spine were feasible and accurate using water-fat-separated images as well as SW-like images with CT-like contrast, both derived from the sUTE-Dixon technique. This was possible by developing a new methodology which solved the smoothness-constrained non-linear inverse water-fat problem while at the same time removing the unwanted low-frequency phase terms. Simultaneously extracting water-fat images and CT-like images from one single MR sequence could be highly useful for clinical examinations due to a reduction of overall examination times and radiation exposure.

## Abbreviations

CT: Computer tomography
DESS: Dual echo steady state
FOV: Field of view
MRI: Magnetic resonance imaging
STIR: Short-tau inversion recovery sequence
sUTE: Single ultrashort echo time sequence
SWI: Susceptibility-weighted images
T1w GRE: T1-weighted gradient-echo sequence
TE: Echo time
TR: Repetition time
ZTE: Zero echo time

## Declarations

### Guarantor

The scientific guarantor of this publication is Georg C. Feuerriegel, MD.

### Statistics and biometry

No complex statistical methods were necessary for this paper.

### Methodology

• retrospective
